# A novel non-fluorescent excited state intramolecular proton transfer phenomenon induced by intramolecular hydrogen bonds: an experimental and theoretical investigation

**DOI:** 10.1038/srep19774

**Published:** 2016-01-21

**Authors:** Hang Yin, Hui Li, Guomin Xia, Chengyan Ruan, Ying Shi, Hongming Wang, Mingxing Jin, Dajun Ding

**Affiliations:** 1 Institute of Atomic and Molecular Physics, Jilin University, Changchun 130012, China; 2 Institute for advanced study, Nanchang University, Nanchang, 330031, China

## Abstract

Two molecules, 1-hydroxypyrene-2-carbaldehyde (HP) and 1-methoxypyrene-2-carbaldehyde (MP) were explored. We investigated their photophysical properties, using experimental transient absorption and theoretical density functional theory/time-dependent density functional theory (DFT/TDDFT). HP and MP have similar geometric conformations but exhibit entirely different photophysical properties upon excitation into the S_1_ state. In contrast to traditional excited state intramolecular proton transfer (ESIPT) in molecules that exhibit either single or dual fluorescence, HP has an unusual non-fluorescent property. Specifically, the ultrafast ESIPT process occurs in 158 fs and is followed by an intersystem crossing (ISC) component of 11.38 ps. In contrast to HP, MP undergoes only an 8 ps timescale process, which was attributed to interactions between solute and solvent. We concluded that this difference arises from intramolecular hydrogen bonds (IMHBs), which induce drastic structural alterntion upon excitation.

Compounds that undergo excited state intramolecular proton transfer (ESIPT) have attracted attention^1^,^2^,^3^,^4^,^5^,^6^ as, for fluorescent probes^7^,^8^,^9^, for use in bioimaging^10^, as light-emitting materials^11^, as photostabilizers^12^, and in photophysical studies^13^,^14^,^15^,^16^,^17^. The characteristic photophysical feature of a molecule undergoing ESIPT is the sub-picosecond timescale and large Stokes shift, which suggest that the structural geometry of the emissive excited state is significantly displaced from that of the ground state^11^,^17^,^18^,^19^.

Many functional organic molecules exhibiting ESIPT have been investigated^19^,^20^,^21^,^22^,^23^,^24^. Furthermore, numerous studies have demonstrated that the ESIPT process involves a heterocyclic ring containing a hydroxyl-group and a neighbouring proton acceptor that can form IMHBs^22^,^25^. Recently, it was found that intramolecular hydrogen bonding plays a crucial role in the proton transfer process^26^. The ESIPT process can be facilitated by the excited-state intramolecular hydrogen bond strengthening in the S_1_ state^26^,^27^. The first ESIPT investigation, which was performed on methyl salicylate (MS) by Weller^28^, demonstrated that MS is a three energy level system containing the ground state, a normal excited state, and a tautomer excited state, and it is the tautomerism that produces dual fluorescence.

However, despite numerous investigations since that of Weller, the ESIPT process still presents challenges both theoretically and experimentally due to the intrinsically complex physical and chemical nature, such as the cleavage and formation of hydrogen bonds and the subsequent nuclear rearrangement with inversion. More recently, experiments by Mitra *et al.*^25^ showed that the ultrafast ESIPT of 4-methyl-2,6-diformyl phenol occurs at approximately 200 fs in cyclohexane followed by an intramolecular vibrational redistribution (IVR) process for approximately 2.8 ps before emitting fluorescence from the ESIPT state. Tang *et al.*^19^ investigated the ESIPT process of 7-hydroxy-1-indanone and its derivatives by theoretical and experimental methods, elaborating the details of the synthesis, photophysical properties, and OLED application. In our previous report^26^, we theoretically investigated the salicylaldehyde (SA) molecule, which has exclusively intramolecular O–H⋅⋅⋅O hydrogen bonds that form a strong quasi-aromatic chelating ring. We found that the hydrogen bonded quasi-aromatic chelating ring in the excited state becomes smaller, which facilitates the ESIPT process. We also discovered that SA exhibits single fluorescence, which unlike the mechanism described by Weller^28^. We believe that the ESIPT system under the effect of IMHBs can exhibit non-fluorescence in addition to single and dual fluorescence. Therefore, we focused on the ESIPT system HP, which was found to be non-fluorescent in the S_1_ state. We also investigated MP molecule, which cannot form IMHBs, for comparison.

The six-membered, intramolecularly hydrogen-bonded system HP is an ideal model to explore the effect of IMHBs on the ESIPT system. To shed light on the ESIPT dynamics of HP, we report a combined experimental and theoretical method to investigate the HP and MP molecules and to confirm experimental results and discover more detailed information.

## Results and Discussion

**Figure 1:**
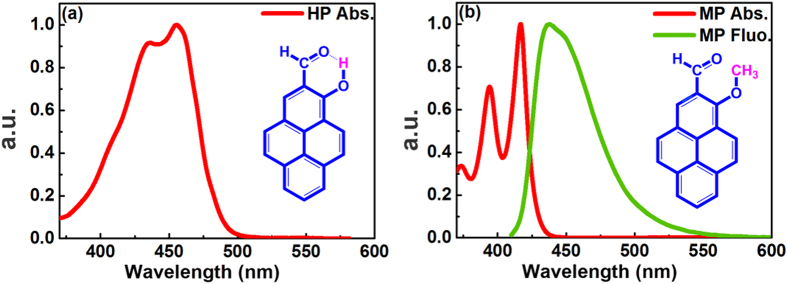
Shapes of steady-state absorption and fluorescence spectra of HP (**a**) and MP (**b**) molecules in CHX. λ_ex_ = 400 nm. Structures of the HP and MP molecules are also shown as an inset in (**a**) and (**b**), respectively.

The structures of the HP (a) and MP (b) molecules are shown in Fig. 1. An IMHB can be formed between the carbonyl group oxygen and the hydroxyl hydrogen in the HP molecule to create a hydrogen-bonded, quasi-aromatic chelating ring. In contrast, the IMHB is not formed in the MP molecule. Figure 1 also depicts the steady-state absorption and fluorescence spectra of HP (a) and MP (b) in cyclohexane (CHX). The absorption spectra show different vibronic structures between HP and MP, which indicates that the maxima of the absorption bands of HP and MP are located near 455 and 417 nm, respectively. The width of the absorption band for HP is broader than for MP, and the MP band is split more than that of HP. Because the two molecules have similar geometric conformations, we suggest that the obvious difference in absorption is caused by IMHBs. The fluorescence peak of MP is at 438 nm. Upon 400 nm excitation, HP is non-fluorescent, which is different from dual-fluorescent MS investigated by Weller^28^.

**Figure 2:**
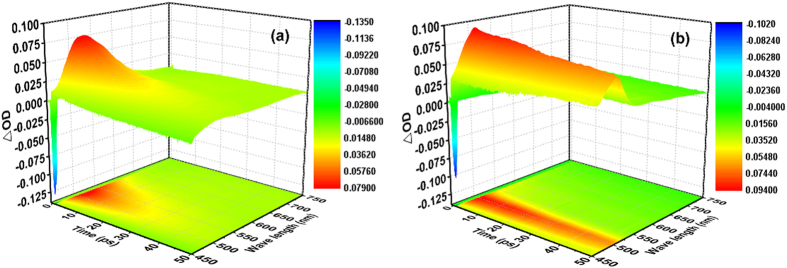
3D image plots of the transient absorption for HP (**a**) and MP (**b**) in CHX after excitation at 400 nm.

We performed femtosecond transient absorption spectroscopy to investigate the excited state properties of HP and MP in CHX. Figure 2 depicts the 3D image plot of transient absorption for HP and MP as a function of wavelength and time delay after excitation. For HP (Fig. 2a), there are two main components, absorption (positive signal) at approximately 520 nm and emission (negative signal) at approximately 450 nm. For MP (Fig. 2b), the absorption is at approximately 500 nm, and the emission is at approximately 460 nm. However, in contrast to HP, the lifetime of the absorption at approximately 500 nm is relatively longer, and the absorption width is relatively narrower for MP. The 3D plot shows a global overview of the excited dynamics of HP and MP.

**Figure 3:**
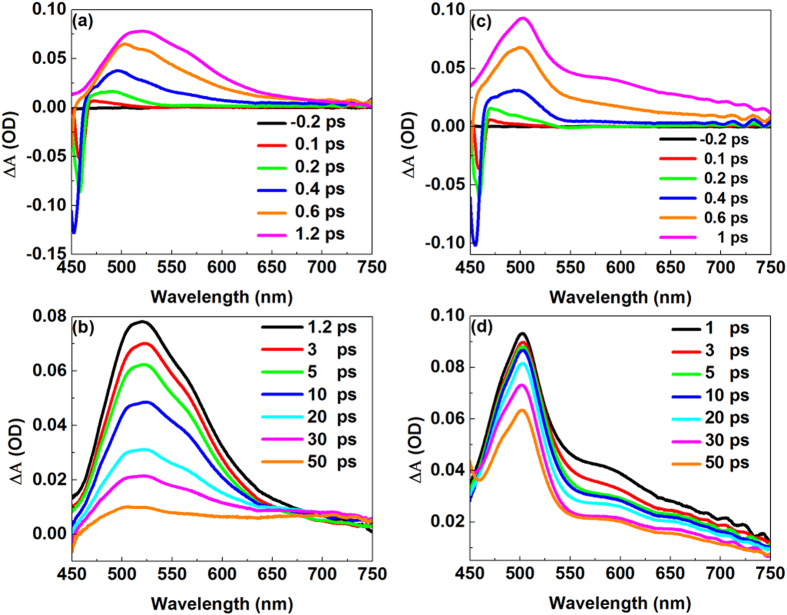
Femtosecond transient absorption spectra recorded in CHX for HP between −0.2 and 1.2 ps (**a**), from 1.2 to 50 ps (**b**), and for MP between −0.2 and 1 ps (**c**), from 1 to 50 ps (**d**).

The transient positive and negative differences in absorbance values, which evolve over delay time after excitation at 400 nm, are shown in Fig. 3. Figure 3(a,b) depict the transient absorption spectra of the HP molecule at different delay times. Initially, an emission band at approximately 455 nm appears and ends within hundreds of femtoseconds with a slight blue-shift. In the meantime, a broad absorption band appears, and the shape of the transient absorption spectra is changed with time. Before 1.2 ps, the characteristic peak of the absorption band has an obvious red shift from 470 nm to 520 nm, and the intensity and the width of the absorption band increase with time. After 1.2 ps, the intensity and width of the absorption band begin to decrease, and the characteristic peak has a slight blue-shift from 520 nm to 502 nm that evolves over delay time. In contrast, the time evolution of the transient absorption spectra of MP is shown in Fig. 3(c,d). It takes less time to reach the maximum intensity of absorption, and the width is narrower for MP than HP at the same delay time. Moreover, in the range of 0 to 1 ps, the characteristic peak absorption band has a relatively smaller red-shift from 470 nm to 502 nm compared to that of HP. The rate of the absorption intensity decrease is much slower than that of the HP molecule and is without the blue-shift of the characteristic peak.

To investigate the lifetime of the dynamics processes for HP and MP, a global fit analysis was used, in which sets of kinetic curves were fit simultaneously using exponential components with common lifetimes (Table 1). The kinetic curves for HP were fitted to two exponentials with lifetimes τ_1_ ~ 158.6 fs and τ_2_ ~ 11.38 ps. In contrast to HP, the single index model provided better fits to the experimental data of MP, and the lifetime τ_1_ is 8.42 ps.

Through our experimental investigation, we explored how the HP molecule is different from the MP molecule. The HP molecule exhibits a dual ultrafast process and non-fluorescence emission during excitation at 400 nm, but MP exhibits just one ultrafast process and fluorescence emission. To reveal the mechanism of this phenomenon, we performed theoretical analyses using DFT and TDDFT.

Table 2 shows the calculated absorption and emission peaks of the HP and MP molecules using the TDDFT/B3LYP/TZVP method. The absorption spectrum is calculated by vertical excitation from the ground state to the S_1_ state, and the emission spectrum is calculated by emission from the S_1_ state equilibrium geometry to the ground state non-equilibrium geometry. The theoretical results are in good agreement with the experimentally measured absorption and emission maxima, which suggests that the method is effective and credible. Moreover, the small Stokes shift of MP suggests that its primary de-excitation process is fluorescence.

**Figure 4:**
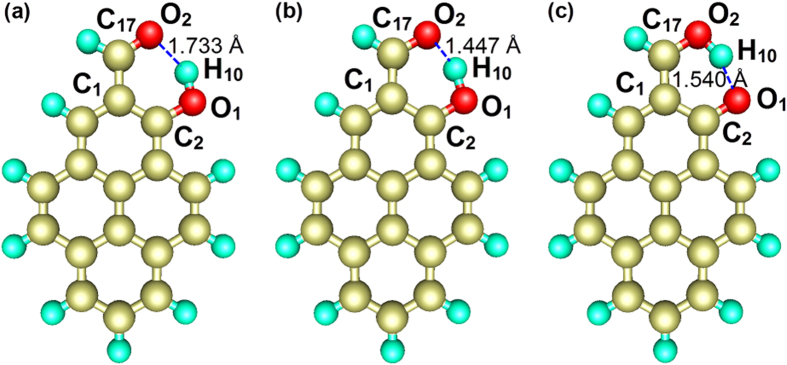
Schematic plots of the HP structure optimized at the S_0_ and S_1_ states in the enol (**a**), enol* (**b**) and keto* (**c**) forms; the length shown in the picture is the length of the hydrogen bond. The numerical values of the key geometric parameters are shown in Table S1. (The golden atom represents C, the light green atom represents H, and red atom represents O. The lengths of the hydrogen bonds are also shown.).

**Figure 5:**
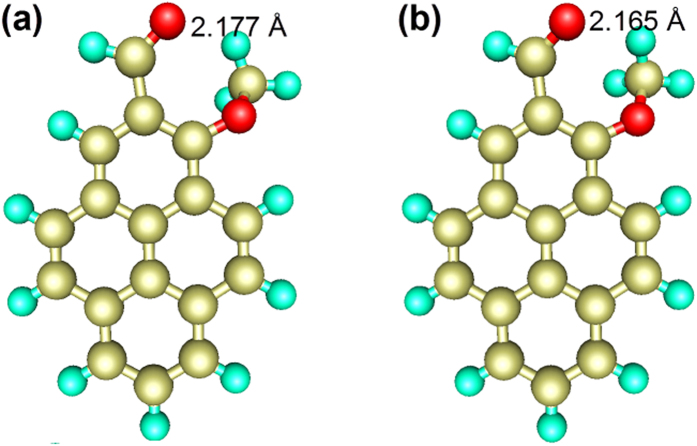
Schematic plots of the MP structure optimized at the S_0_ (**a**) and S_1_ states (**b**).

To investigate the mechanism of de-excitation for HP, we optimized the geometric conformations of the enol (S_0_), enol* (S_1_) and keto* (S_1_) forms and the results are shown in Fig. 4. These optimized geometries show that the IMHB of HP is formed between C_17_=O_2_ and O_1_–H_10_ in the enol and enol* form, wherease an IMHB can be formed between C_2_=O_1_ and O_2_–H_10_ in the keto* form. In addition, the numerical values of the key geometric parameters of different electronic states can be found in supplementary Table S1. Based on the results of the geometric conformations, we found that HP exhibits an ESIPT process corresponding to the fs timescale in our experiment^4^. Upon excitation to enol*, the distance between O_2_ and H_10_ decreases from 1.733 Å to 1.447 Å, whereas the bond length between O_1_ and H_10_ increases from 0.988 Å to 1.063 Å, which suggests that H_10_ has the tendency to approach O_2_ and depart from O_1_. Eventually, HP evolves into the keto* state. The intramolecular C_17_=O_2_⋅⋅⋅H_10_–O_1_ hydrogen bond breaks, concomitantly with the formation of an intramolecular C_2_=O_1_⋅⋅⋅H_10_–O_2_ hydrogen bond. The keto* state bond angles C_17_C_1_C_2_ and C_1_C_2_O_1_ are both smaller than the corresponding ground state bond angles, and the keto* state distance between O_1_ and O_2_ becomes shorter than the corresponding ground state distance, which indicates that the hydrogen-bonded quasi-aromatic chelating ring becomes smaller after excitation. Moreover, we found that the IMHB becomes short and that H_10_ transfers from O_1_ to O_2_. The interactions of involved atoms increase when the distances between atoms decrease. Compared to HP, MP cannot form IMHBs in the ground state or in the S_1_ state due to the lack of a hydrogen donor, such as the hydroxyl hydrogen atom of HP, as shown in Fig. 5. Therefore, we concluded that intramolecular hydrogen bonding in the HP molecule facilitates the ESIPT process. In contrast to the complexity of HP, the geometric conformation of MP is much simpler. There is no obvious structural alteration between S_0_ and S_1_, which indicates that MP molecules do not exhibit specific ultrafast processes such as ESIPT and twisted intramolecular charge transfer (TICT), during the de-excitation.

**Figure 6:**
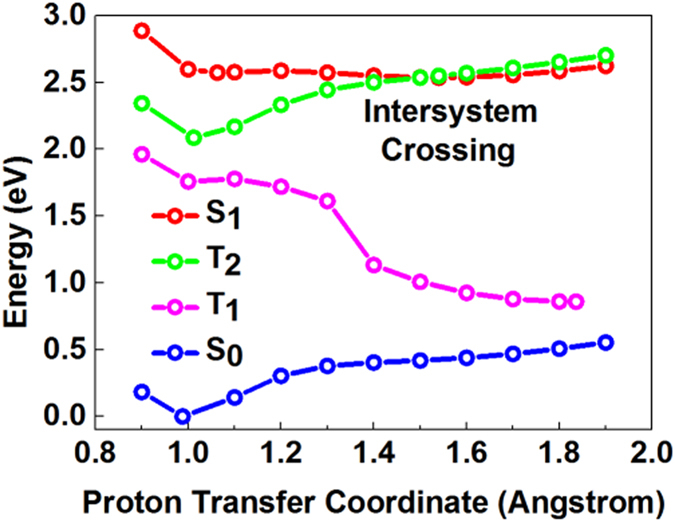
Calculated potential energy curves along the proton transfer coordinate (O_2_─H_2_) of HP in different electronic states.

To demonstrate the details of the ESIPT process of HP and to explore the non-fluorescent mechanism of HP upon excitation to the S_1_ state, we calculated potential energy curves in different electronic states. The potential energy curves of HP were optimized by fixing the distance between O_1_ and H_10_ (proton transfer coordinate) at different values, which are recorded in Fig. 6. There is only one stable geometry in the S_0_ state, which is associated with the enol form. The potential energy curves reveal that the stable points in the S_1_ state correspond to the coordinates 1.063 Å (enol* form) and 1.540 Å (keto* form), respectively. HP has two stable equilibrium geometries after Frank-Condon transition to the S_1_ state. To achieve the equilibrium geometry of the S_1_ state tautomer form, H_10_ transfers from O_1_ to O_2_ and forms the new hydrogen bond C_2_=O_1_⋅⋅⋅H_10_—O_2_. The existence of the S_1_ state tautomer equilibrium geometry is direct evidence of an ESIPT process with photoexcitation to the S_1_ state. The calculation of the electronic excitation energy of HP shown in Table 3 indicates that the energy of the 1^st^ and 2^nd^ triplet states (T_1_ and T_2_) are lower than that of the S_1_ state, and the energy of the 3^rd^ triplet state (T_3_) is higher than that of the S_1_ state. Therefore, we also calculated the potential energy curves of the T_1_ and T_2_ states, as shown in Fig. 6. There is no cross point between S_1_ and T_1_. The occurrence of a cross between S_1_ and T_2_ is at approximately 1.5 Å to 1.6 Å, which corresponds to the coordinate of the S_1_ keto* state, i.e., the ISC process occurs between the S_1_ and T_2_ states. Therefore, combined with the non-fluorescent excitation to the S_1_ state, we concluded that the relaxation pathway of HP after ESIPT is the ISC process from the S_1_ keto* to the T_2_ state^29^,^30^,^31^. Comparing with the results of Weller’s^28^, we can find that both HP and MS have two stable equilibrium geometries after Frank-Condon transition to the S_1_ state. Differently, MS exhibits stepwise dual fluorescence from two stable equilibrium geometries of the S_1_ state, and HP is non-fluorescent due to the ISC process from the S_1_ keto* state to the T_2_ state after the ESIPT process.

**Figure 7:**
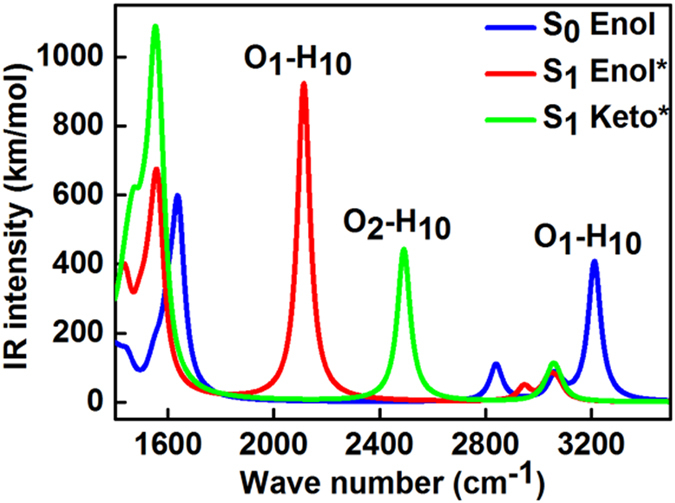
Calculated IR spectra of HP in the S_0_ and S_1_ states (normal form and tautomer form).

The vibrational frequencies of the stretching vibrations of C=O and O–H groups that are involved in hydrogen bonds can provide a specific signature of the hydrogen-bonding dynamics^32^. In the present work, we calculated the IR spectra (B3LYP/TZVP, scaling factor 0.9630^33^) of HP at the S_0_, S_1_ states (enol* and keto* forms). The calculated IR spectra of the ground state and S_1_ state in the spectral range from 1400 to 3500 cm^−1^ are shown in Fig. 7, which contains not only the characteristic peaks of the O_1_–H_10_ group of the S_0_ and S_1_ normal forms but also the characteristic peaks of the O_2_–H_10_ group of the S_1_ tautomer form. For the S_1_ tautomer form, the characteristic peaks at approximately 3213 cm^−1^, which is assigned as ν(O_1_–H_10_) in S_0_, and at approximately 2113 cm^−1^, which is assigned as ν(O_1_–H_10_) in the enol* form, disappeared, whereas a new characteristic peak appeared at approximately 2492 cm^−1^ and was assigned as ν(O_2_–H_10_). Thus, we can conclude that the O_1_–H_10_ bond breaks, and a new O_2_–H_10_ bond forms, just as the geometry optimization shows in Fig. 4. Consequently, as auxiliary evidence, the IR spectra correlate well with the result that H_10_ transfers from O_1_ to O_2_ in the S_1_ excited state. Moreover, corresponding IR spectra of HP at the triplet state can be seen in supplementary Figure S6.

**Figure 8:**
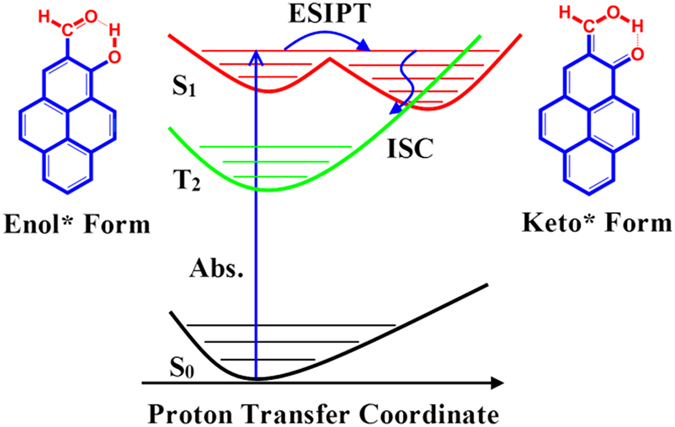
Schematic view of the de-excitation mechanism of HP.

A comparison between the experimental and theoretical results allowed us to understand the properties of the systems more deeply, and the theoretical method gave the experimental results a clear interpretation. We show the mechanism of de-excitation for HP in Fig. 8. First, the HP molecule in the S_0_ enol state is excited to the S_1_ enol* state; subsequently, an ESIPT process from the S_1_ enol* state to the S_1_ keto* state occurs at a timescale of approximately 158.6 fs. The ISC between S_1_ and T_2_ in approximately 11.38 ps gives a reasonable explanation for the non-fluorescence of HP upon excitation into the S_1_ state. In Fig. 3(a,b), the negative signals below 460 nm for HP molecules correspond primarily to ground state depletion. In the range from 0 to 0.1 ps, because the S_1_ keto* state is not formed, the characteristic peak at approximately 470 nm is attributed to the S_1_ enol* state. From 0.1 to 1.2 ps, the observed redshift and intensity increment of the absorption band at approximately 520 nm suggest that the population of the S_1_ keto* state increases with the relaxation from the hot S_1_ keto* to the cold S_1_ keto* state. Between 1.2 and 10 ps, the intensity decrement of the absorption peak at approximately 520 nm arises from the decrement of the population in the S_1_ keto* state due to the ISC process from S_1_ keto* to T_2_ on an approximately 10 ps timescale. This process induces the non-fluorescent property of HP upon excitation to the S_1_ state. After 10 ps, only the intensity and not the shape of the absorption band changes, thus; the characteristic peak at approximately 502 nm corresponds to the T_2_ state. Compared with the HP molecule, the de-excitation of MP is simpler, as shown in Fig. 3(c,d). Before 1 ps, the observed slight redshift and intensity increment of the absorption at approximately 502 nm suggest that the population of the S_1_ state increases with the relaxation from the hot S_1_ to the cold S_1_ state. After 1 ps, the dominant de-excitation process is fluorescence, which is a ns timescale process. Therefore, the lifetime of S_1_ for MP is much longer than that for the HP molecule. Furthermore, the lifetime of the HP τ_1_ was attributed to the ESIPT process, and τ_2_ was associated with the ISC process. The lifetime of the MP τ_1_ corresponded to interactions between solute and solvent.

## Conclusion

In the present exploration, femtosecond transient absorption spectroscopy and DFT/TDDFT were performed to investigate the photoexcitation of HP and MP. We demonstrated that HP exhibits the ESIPT process in the S_1_ state and discovered the reason for the non-fluorescenec of HP upon excitation to the S_1_ state in CHX. Specifically, the ESIPT process happens in 158 fs, whereas the ISC process occurs on an 11.38 ps timescale. In contrast to HP, MP undergoes an 8 ps timescale process, which was attributed to interactions between the solute and solvent in the S_1_ state, and it exhibits fluorescence upon excitation to the S_1_ state. The geometric conformations of HP and MP are similar, but HP differs most from MP in that it is able to form IMHBs. We concluded that the ESIPT and the ISC of HP arise from the existence of IMHBs, which induce drastic structural alterations between the S_0_ and S_1_ state.

## Methods

In the present work, the ground-state and electronic excited-state geometry optimizations were performed by the DFT and TDDFT^34^,^35^,^36^,^37^,^38^,^39^,^40^,^41^,^42^ methods, respectively. The B3-LYP (Becke’s three-parameter hybrid exchange function with Lee-Yang-Parr gradient-corrected correlation) functional and the TZVP basis sets (the triple-ζ valence quality with one set of polarization functions) were used in our DFT and TDDFT calculations^43^,^44^,^45^,^46^. In addition, the IR intensities were derived from the gradients of the dipole moment. The electronic structure calculations were performed using the TURBOMOLE program suite^45^,^47^.

HP and MP were synthesized according to the literature^48^. The cyclohexane (CHX) was a super-dry, spectrum-pure, quality reagent purchased from J&K (China) and was used without further purification. The concentration of HP and MP in CHX was 5*10^-6^ mol/L.

The steady-state absorption and fluorescence spectra were measured with a UV 2550 UV-VIS spectrophotometer and a RF5301 fluorescence spectrophotometer (Shimadzu), respectively.

Transient absorption imaging was based on a femtosecond laser (Coherent Libra, US), which was used as the light source with a power of approximately 4W at 1 kHz repetition rate and a wavelength of 800 nm where the full width at half maximum (FWHM) was 50 fs. The fundamental laser was separated into two beams in a ratio of 9:1. The more intense beam was used for generating the second harmonic (λex = 400 nm) of the fundamental laser using a 0.5 mm BBO (β-BaB2O4, Fujian Castech Crystals Inc., China), which was applied as a pump laser for exciting samples. The energy per pump pulse at the sample was approximately 3 μJ. The other beam passed through a controlled delay line and then focused into a sapphire plate to generate sub-picosecond, super-continuum white light, which served as the probe laser. Two laser beams were incident on the sample in a 0.5 cm quartz cuvette at a small angle (θ ≤ 5°). The fused quartz sample cell was placed in the beam path, and the beam diameter was 2 mm. The sensitivity range was 450–750 nm. All experiments were performed at room temperature (22 °C).

## Additional Information

**How to cite this article**: Yin, H. *et al.* A novel non-fluorescent excited state intramolecular proton transfer phenomenon induced by intramolecular hydrogen bonds: an experimental and theoretical investigation. *Sci. Rep.* **6**, 19774; doi: 10.1038/srep19774 (2016).

## Supplementary Material

Supplementary Information

## Figures and Tables

**Table 1:** Transient absorption kinetics lifetimes of HP and MP in CHX solvent by global analysis.

System	τ_1_ (fs)	τ_2_ (ps)
HP	158.6 ± 19.12 (12.06%)	11.38 ± 2.859 (25.12%)
MP	—	8.42 ± 0.765 (9.09%)

The meaning of the proportion is the deviation percentage.

**Table 2:** Calculated absorption peak and emission peak of the HP and MP molecules compared with the experimental data (unit: nm).

System	Abs. (Thero.)	Abs. (Exp.)	Fluo. (Thero.)	Fluo. (Exp.)
HP	442	455	─	─
MP	405	417	430	438

**Table 3:** The electronic excitation energy (E) corresponding to the oscillator strength (OS) for low-lying electronically excited states of HP.

	E (nm)	OS
T_3_	381	0.018
S_1_	442	0.052
T_2_	544	0.270
T_1_	600	0.989
