# Supplementary material for: A novel non-fluorescent excited state intramolecular proton transfer phenomenon induced by intramolecular hydrogen bonds: an experimental and theoretical investigation
Source: Sci Rep. 2016 Jan 21;6:19774. doi: 10.1038/srep19774 (PMC4726414; doi:10.1038/srep19774)
Supplement: Supplementary Information [file srep19774-s1.doc]

**Supporting Information**

**A novel non-fluorescent ESIPT phenomenon induced by intramolecular hydrogen bond: an experimental and theoretical investigation**

Hang Yin, Hui Li, Guomin Xia, Chengyan Ruan, Ying Shi*, Hongming Wang*, Mingxing Jin and Dajun Ding^*^

**1. Synthesis**

**1-methoxypyrene-2-carbaldehyde (MP)**

Compound MP ^1^H NMR (400 MHz, *CDCl_3_*) δ ppm 10.82 (s, 1H), 8.61 (s, 1H), 8.40 (d, *J =* 9.25 Hz, 1H), 8.24-8.09 (m, 3H), 8.09-7.98 (m, 3H), 4.24 (s, 3H); ^13^C NMR (400 MHz, *CDCl_3_*) δ ppm 190.54 (s,1C), 132.09 (s,1C), 131.90 (s,1C), 128.28 (s,1C), 127.84 (s,1C), 127.82 (s,1C), 127.61 (s,1C), 127.49 (s,1C), 125.55 (s,1C), 125.43 (s,1C), 123.97 (s,1C). 123.95 (s,1C), 121.05(s,1C), 117.70(s,1C), 117.389(s,1C), 114.52(s,1C), 65.83(s,1C).

**1-hydroxypyrene-2-carbaldehyde (HP)**

Compound HP ^1^H NMR (400 MHz, *CDCl_3_*) δ ppm 10.20 (s, 1H), 8.43 (d, *J =* 9.09 Hz, 1H), 7.86-7.74 (m, 2H), 8.12-7.96 (m, 5H), 11.86 (s, 1H); ^13^C NMR (400 MHz, *CDCl_3_*) δ ppm 197.23 (s,1C), 155.84(s,1C), 133.04(s,1C), 132.61(s,1C), 129.45(s,1C),128.51 (s,1C), 127.95 (s,1C), 127.21 (s,1C), 127.07 (s,1C), 125.80 (s,1C), 124.88 (s,1C), 124.77 (s,1C), 124.54(s,1C), 124.19(s,1C),121.40(s,1C), 119.58(s,1C), 116.85(s,1C).

Figure S1. ^1^H NMR spectra of compound 1-methoxypyrene.

Figure S2. ^1^H NMR spectra of compound MP.

Figure S3. ^13^C NMR spectra of compound MP.

Figure S4. ^1^H NMR spectra of compound HP.

Figure S5. ^13^C NMR spectra of compound HP.


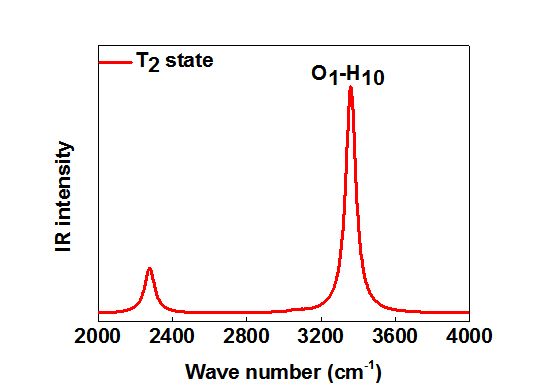


Figure S6 Calculated IR spectra of HP in the T_2_ state.

**3. Relevant results**

**Geometry Optimization**

|  | C_1_–C_2_ | C_2_–O_1_ | C_1_–C_17_ | C_17_–O_2_ | O_1_–O_2_ | O_2_–H_10_ | O_1_–H_10_ | C_17_C_1_C_2_ | C_1_C_2_O_1_ |
| --- | --- | --- | --- | --- | --- | --- | --- | --- | --- |
| Enol | 1.415 | 1.340 | 1.452 | 1.229 | 2.617 | 1.733 | 0.988 | 120.15 | 121.32 |
| Enol* | 1.435 | 1.316 | 1.453 | 1.281 | 2.458 | 1.447 | 1.063 | 118.80 | 118.84 |
| Keto* | 1.448 | 1.274 | 1.422 | 1.318 | 2.504 | 1.034 | 1.540 | 119.33 | 120.23 |

Table S1. Calculated bond lengths (Å) and bond angles (deg) of the HP molecule in different electronic states.

**Calculated the ESIPT reaction rate**

To validate the reliability of our experiment result that the lifetime of the ESIPT process in the HP molecule, we simulated the reaction rate using the follow formula^[1]^.

$$k=\frac{k_{B}T}{h}\exp(\frac{-\Delta G}{RT})$$

In the formula, k is the rate we want to calculate, K_B_ is the Boltzmann constant, h is the Planck constant, R is the universal gas constant, $\Delta G$ is the activation free-energy, and T is the temperature we have chosen. There into, we calculated the difference between the energy of S_1_ Enol* and the energy of transition state in ESIPT process at the S_1_ state to get $\Delta G$. T is our experiment temperature 22℃ (295.15 K). We calculated that k=3.7068*10^12^ s^-1^. Therefore, τ=1/k=269.78 fs. The result is very close our experimental data which is 158.6 fs.

**References**

[1] R. D. Levine*, Molecular reaction dynami*cs, Cambridge University Press**, 20**05.
